# YOLO POD: a fast and accurate multi-task model for dense Soybean Pod counting

**DOI:** 10.1186/s13007-023-00985-4

**Published:** 2023-01-28

**Authors:** Shuai Xiang, Siyu Wang, Mei Xu, Wenyan Wang, Weiguo Liu

**Affiliations:** 1 College of Agronomy, Sichuan Agricultural University, 211-Huimin Road, Wenjiang District, Chengdu, 611130 People’s Republic of China; 2 Key Laboratory of Crop Ecophysiology and Farming System in Southwest China (Ministry of Agriculture), Sichuan Engineering Research Center for Crop Strip Intercropping System, Sichuan Agricultural University, Chengdu, 611130 People’s Republic of China

**Keywords:** Soybean, Deep learning, Objection detection, Multi-Task learning, Yield estimation

## Abstract

**Background:**

The number of soybean pods is one of the most important indicators of soybean yield, pod counting is crucial for yield estimation, cultivation management, and variety breeding. Counting pods manually is slow and laborious. For crop counting, using object detection network is a common practice, but the scattered and overlapped pods make the detection and counting of the pods difficult.

**Results:**

We propose an approach that we named YOLO POD, based on the YOLO X framework. On top of YOLO X, we added a block for predicting the number of pods, modified the loss function, thus constructing a multi-task model, and introduced the Convolutional Block Attention Module (CBAM). We achieve accurate identification and counting of pods without reducing the speed of inference. The results showed that the R^2^ between the number predicted by YOLO POD and the ground truth reached 0.967, which is improved by 0.049 compared to YOLO X, while the inference time only increased by 0.08 s. Moreover, MAE, MAPE, RMSE are only 4.18, 10.0%, 6.48 respectively, the deviation is very small.

**Conclusions:**

We have achieved the first accurate counting of soybean pods and proposed a new solution for the detection and counting of dense objects.

**Supplementary Information:**

The online version contains supplementary material available at 10.1186/s13007-023-00985-4.

## Introduction

The selection of new crop varieties and the improvement of cultivation management rely heavily on yield tests. For soybeans, the yield is composed of three factors: the number of pods per plant, the number of seeds per pod, and the seed size [1]. As an important factor affecting yield, the number of pods is mainly obtained by manual counting. Manual counting is laborious, time-consuming, and error-prone. Therefore, developing an efficient and accurate pod counting method is of great significance for soybean breeding and cultivation.

Modern breeding requires a large amount of material with different genetic backgrounds, making estimating yield a difficult task. Because of the ease of access to digital images and the rapid development of image technology, digital images are widely used for crop yield estimation. Duan et al. [2] obtained the projected panicle area, projected area of leaf, stem dimension, and fractal dimension from the images, then estimated rice yield through these image features. Zhu et al. [3] used support vector machines to detect wheat ears and thus evaluate the yield. Pranga et al. [4] used UAV to collect RGB and multispectral images and used the Random Forest to accurately estimate the yield of Herbage. Through image processing techniques and machine learning, crop yields can be estimated with high throughput. However, such methods are poorly robust and their accuracy decreases when environmental conditions change.

Deep learning has developed rapidly in recent years. With the increase of model parameters and the continuous optimization of model structure, deep learning can solve more complex tasks and get better performance [5, 6]. Estimating yield by deep learning is highly accurate and robust. Shao et al. [7] used the LC-FCN model to detect and count rice ears, and Wu et al. [8] used image processing techniques and deep learning to count the number of rice grains. Lu et al. [9] proposed TasselNet to detect and count maize tassels. Wang et al. [10] proposed an improved EfficientDet-D0 model for wheat head counting. It is an effective approach to estimating yield by detecting yield organs, and this approach has been successfully applied to many crops.

The soybean yield is highly correlated with the number of pods [11], however, there are few reports on pod identification and counting, and the current methods are not effective. For pod detection and counting, there are two main issues. First, not accurate enough. Compared with maize tassel and wheat head, soybean pods are very dense, with heavy overlap between pods. It is difficult to identify and locate all pods in the images. Riera et al. used RetinaNet for pod detection and counting and the highest correlation was only 0.711 [12]. To achieve accurate detection and counting, it is necessary to remove the pods from the branches to avoid overlapping [13]. Second, not fast enough. Yang et al. used Swin Transformer to identify pods [14]. Although the identification is relatively accurate, due to the large number of parameters of Swin Transformer, the detection speed is very slow and it is difficult to detect in real-time.

Most of the object detection networks are designed for the COCO dataset, which has an average of 7.7 objects per image [15]. While the pods are very dense, the simple use of object detection networks is often not very accurate. In areas where pods overlap significantly, the texture features are significantly different from non-overlapping areas. The complex texture features may suggest that there are more obscured pods in the area. For object detection networks, such information is ignored in order to more accurately identify typical objects. The model may perform better if it can take advantage of the additional information. CLIP uses natural language to enhance the learning of visual concepts, greatly improving the generalization ability of the model [16]. Multi-task learning, due to differences between tasks, can help the model focus on more information. A suitable auxiliary task can help to improve the main task [17]. Through multi-task learning, the model can extract additional information that may alleviate the obscuration of the pods.

YOLO (You Only Look Once) is a series of classical object detection models that balances speed and accuracy, widely used in agriculture. Tian et al. [18] combined the YOLO V3 model with DenseNet and proposed the YOLOV3-density, achieved accurate identification of apples at different growth stages. Yang et al. [19] added a self-attentive module to YOLO V4 to improve the accuracy of counting wheat ears. Ge et al. [20] made a series of improvements to YOLO V5s and proposed YOLO-Deepsort, thus tracking and counting tomatoes at different growth periods. YOLO X is one of the latest achievements of the YOLO series and it performs better than the previous YOLO model [21].

We propose an approach based on the YOLO X framework. We modify the model into a multi-task model by adding a pod number prediction module and modifying the loss function. We have also made a series of improvements to the model to improve performance without sacrificing speed.

## Materials and methods

### Pod counting datasets

**Fig. 1:**
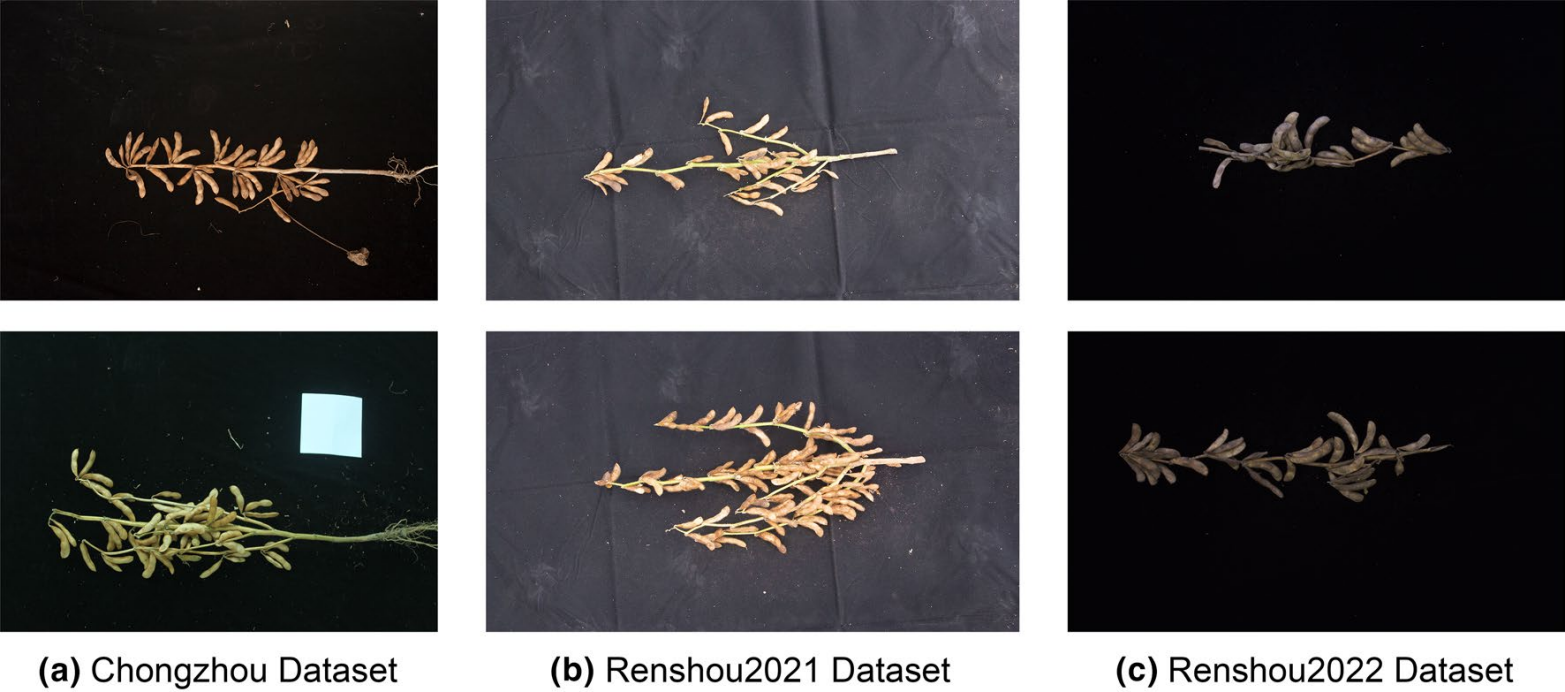
Examples of soybean images in three different datasets

To better validate the generalization ability of the model, we used three datasets in this study. The first dataset is Chongzhou dataset. The field experiment was conducted in 2021 at Sichuan Agricultural University Chongzhou Experimental Base (103.40°E, 30.39°N), with 70 cm row spacing and 20 cm plant spacing. These images were taken by Canon 700D, and the image size was 4752 × 3168 pixels (Fig. 1a), a total of 570 images were acquired. The other two datasets are Renshou2021 dataset and Renshou2022 dataset, and they were obtained from Renshou Farm of Sichuan Agricultural University (104°08′E, 29°59'N). Field experiments for Renshou2021 and Renshou2022 datasets were conducted in 2021, 2022 respectively, the row spacing is 70 cm and the plant spacing is 20 cm. The Renshou2021 dataset was taken by Canon 750D, and the image size was 5184 × 2916 pixels (Fig. 1b), a total of 878 pictures were acquired. The Renshou2022 dataset was taken by Hikvision MV-CH250-90GC, the image size is 3960 × 2392 pixels (Fig. 1c), including 795 images. All pictures were taken under natural light with a black light-absorbing cloth in the background. Depending on the size of the plants, the camera is 120–150 cm above the plant. In Additional file 1, the varieties of soybeans in each dataset are listed, and 5–15 pictures are taken for each variety.

A total of 1448 images from the Chongzhou and Renshou2021 datasets were annotated with LabelImg [22], each pod in the images is annotated with a bounding rectangle. Images from the Chongzhou dataset are used to train the model. Images from the Renshou2021 dataset are used to evaluate the model detection effect, calculate the AP_50_, Precision and Recall. The details of the datasets are given in Table 1. In addition, a total of 1673 images from Renshou2021 and Renshou2022 were used to evaluate the accuracy of pod counting, calculating R2, MAE, MAPE, RMSE.

**Table 1:** The detail of datasets

Dataset name	Country	Location	Acquisition date	Number of images	Image size (Pixels)	Total	Average
Chongzhou	China	Chongzhou, Sichuan	07/2021	570	4752 × 3168	18, 755	32.90
Renshou2021	China	Renshou, Sichuan	10/2021	878	5184 × 2916	53, 895	61.38
Renshou2022	China	Renshou, Sichuan	07/2022	795	3960 × 2392	–	–

The column “Total” represents the total number of pods in the dataset, “Average” indicates the average number of pods in one picture

#### Data augmentation

**Fig. 2:**
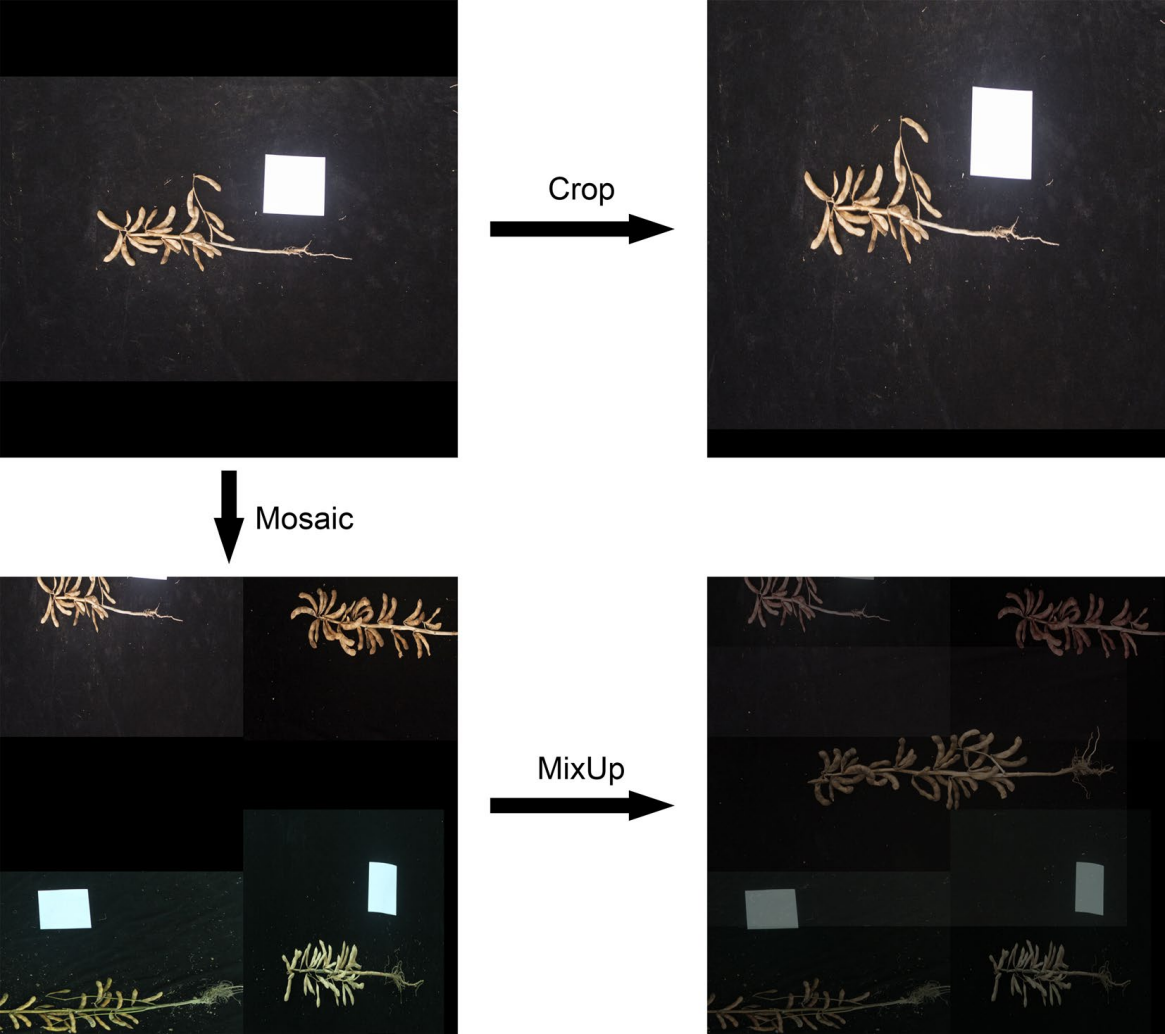
Illustration of YOLO POD's image augmentation pipeline

To enhance the robustness of the model and prevent overfitting, two different data augmentation methods were used. 1) Randomly crop the height of the images. Because the soybean plants are placed horizontally, and most plants are elongated, so there are more background areas on the top and bottom of the image, this part of the background was randomly cropped to help the model detect small objects such as pods. 2) Mosaic [23] and MixUp data augmentation [24]. Randomly select 4 images, after random scaling, mix the 4 pictures, then mix the mixed picture with a new picture. The pipeline for data augmentation is shown in Fig. 2. 

Each iteration randomly uses one of the two data augmentation methods. The probabilities of random cropping, Mosaic, and MixUp are 0.4, 0.6, and 0.5, respectively.

During training, all images of the training set are iterated once in each epoch, and the above random augmentation is applied to each read of each image.

### YOLO POD

To achieve the full potential of our solution, we needed to choose an architecture suitable for detecting soybean pods. YOLO (You Only Look Once) series models are highly accurate and fast. YOLO X [21] is one of the latest achievements of the YOLO series, featuring an anchor-free design. The location, size and orientation of pods are variable, so the anchor free design can better identify dense pods. Therefore, we chose YOLO X as our baseline and built on top of it.

**Fig. 3:**
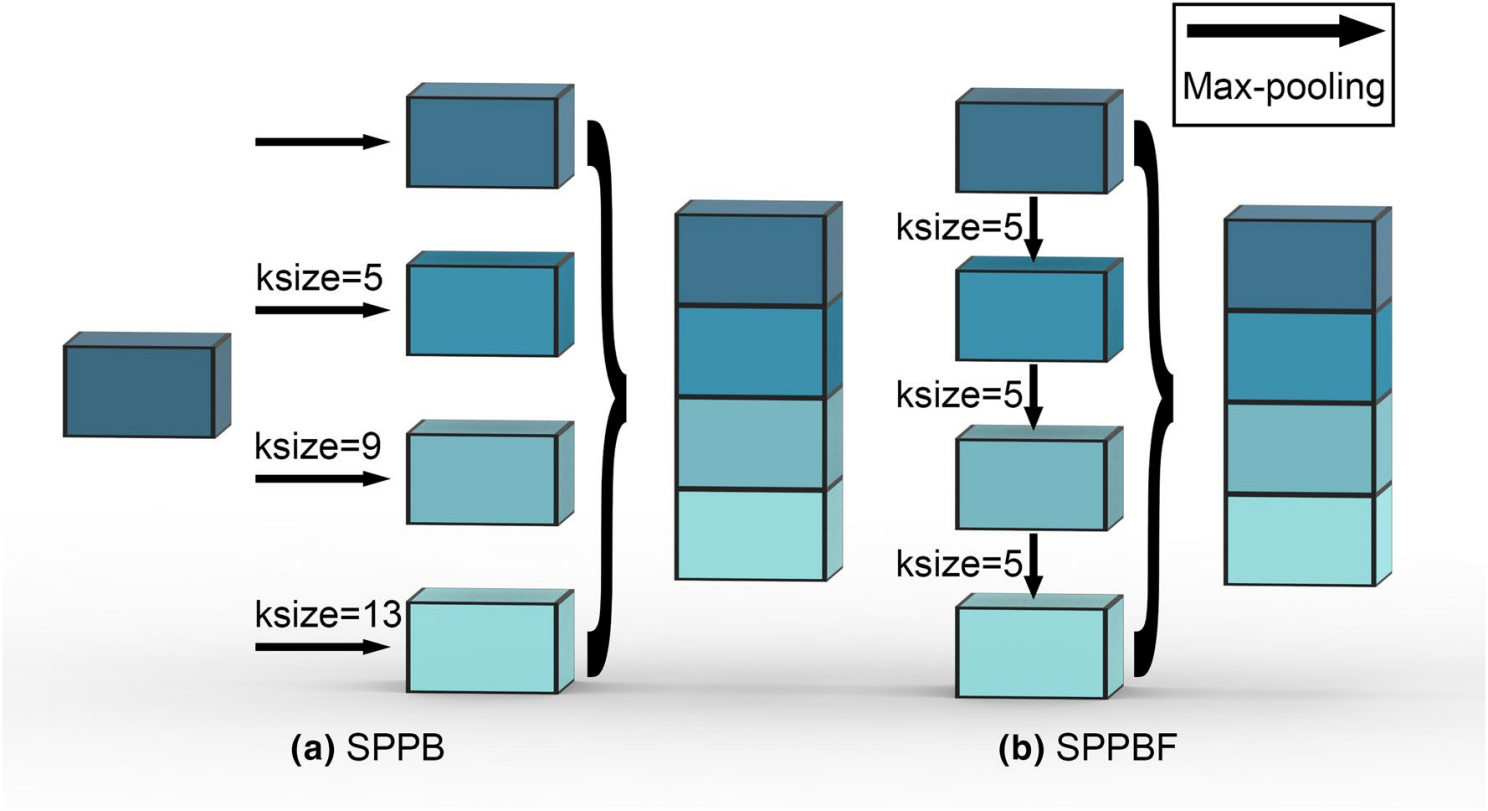
Illustration of the overall structure and sub-modules of YOLO X

The structure of YOLO X is shown in Fig. 3. YOLO X uses CSPNet [25] to extract features, PANet [26] to fuse features, and finally uses two sets of decoupled heads for classification and regression, the IoU branch is added on the regression branch.

Based on YOLO X, we mainly modified the YOLO head. We adopted three sets of decoupled heads to process features at different scales for classification, localization, and prediction of the number respectively, added the self-attention structure, and improved the loss function. In addition, we employed mixed precision training, adopted the SPPF structure.

#### Mixed precision

Mixed Precision Training [27] is a technique that uses both single precision and half-precision when training. It can greatly reduce memory consumption and accelerate the training of the model.

We use mixed precision to reduce the memory consumption of the model, thus using the larger batch size and image size in training, and speeding up training.

#### Spatial pyramid pooling-fast

**Fig. 4:**
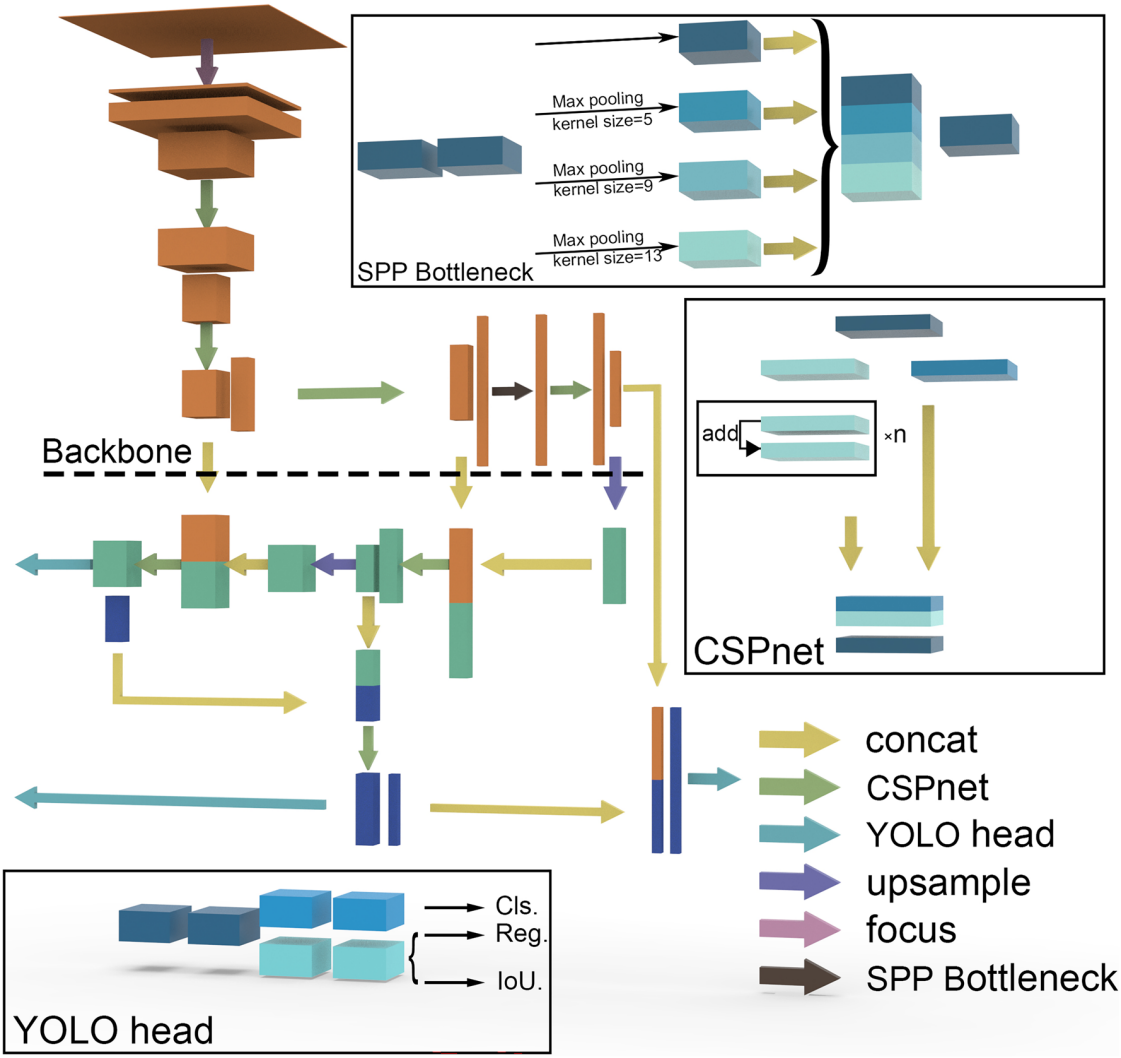
Illustration of the difference between Spatial Pyramid Pooling and the Spatial Pyramid Pooling-Fast. SPPB uses 3 pooling layers with different kernel-size, while SPPBF uses 3 consecutive pooling with kernel-size = 5

SPP (Spatial Pyramid Pooling) [28] can effectively expand the perceptual field of the model and enhance the robustness of the model. However, SPP needs to repeat the maximum pooling four times for a feature map (Fig. 4a), the feature maps obtained by max-pooling are not fully used, which takes up a lot of memory and runs slowly. Therefore, Jocher proposed the SPPF (Spatial Pyramid Pooling-Fast) [29], which performs maximum pooling of feature maps sequentially (Fig. 4b), reducing memory usage and improving running speed. We replace SPP with SPPF.

Furthermore, we replace the Focus block with a convolutional layer with kernel size = 6 and stride = 2, they are computationally equivalent [29].

#### Self-attention based YOLO head

**Fig. 5:**
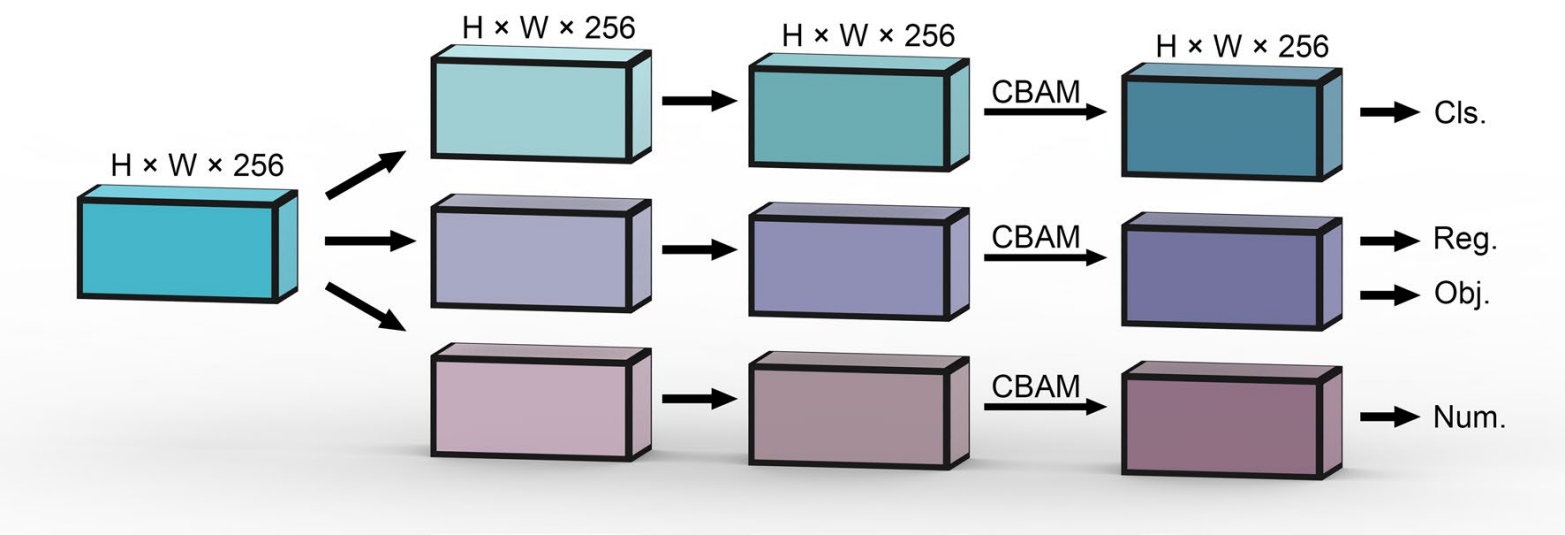
Illustration of YOLO POD's detection head. On top of YOLO X head, we added the self-attention module and a new branch for predicting the number of pods

The images we collected have less pod area and more background (Fig. 2), while the background does not help to count the pods, so to reduce the effect of the background and let the model focus on the pods, we introduced CBAM (Convolutional Block Attention Module) [30] in the YOLO head (Fig. 5).

CBAM is a lightweight and general module, including spatial attention and channel attention. The weights of different regions and channels are calculated by average pooling, max pooling, and a simple fully connected layer. It makes the model focus on the important information.

#### Number prediction module

To help the model learn information reflecting the number of pods, the Number Prediction Module was added to the YOLO head. The behavior of predicting numbers differs greatly from the behavior of identification and localization, in order to avoid the prediction of numbers directly affecting the identification of pods, while enhancing the ability of the backbone to extract number information, as shown in Fig. 5, the Number Prediction Module is decoupled from the module for classification and localization.

We think that the information reflecting the number of pods is more spatially relevant, so we compress the channels to 1, flatten it, and then use a fully connected layer to fuse the information of different scales.

The Number Prediction Module is designed to help the network learn additional information to improve the performance of the model. It serves for training, so there is no need to call this module in inference, which can speed up the inference.

#### Number loss function

The output of the Number Prediction Module is the probability of different pod numbers in one image. In our case, the network will predict the probability that the number of pods is 0, 1, 2, 3 ……297, 298, 299.

For the output of the network, it is first normalized by Softmax to get a smoother probability distribution. The formula is defined as follows:

1 $$Soft\,max\left({x}_{i}\right)=\mathit{log}\left(\frac{\mathit{exp}\left({x}_{i}\right)}{{\sum }_{n=1}^{N}exp\left({x}_{n}\right)}\right)$$

x_i_ = Probability that i is the actual number of pods

N = 300

For the result after Softmax, Negative Log Likelihood Loss is calculated. Here, the number of bounding boxes in a picture is used as the ground truth. Loss is defined as:

2 $${\mathcal{L}}_{num}\left(x,y\right)={\sum }_{b=1}^{B}\frac{-{w}_{yb}{x}_{b,yb}}{{\sum }_{b=1}^{B}{w}_{yb}}$$

B = Batch size

w_yb_ = 1, weight

x_b,yb_ = predicted confidence for ground truth

The total loss is summed by the number loss and YOLO loss:

3 $${\mathcal{L}}_{total}={\lambda }_{num}{\mathcal{L}}_{num}+{\lambda }_{YOLO}{\mathcal{L}}_{YOLO}$$

λ_num_ = 0.3, λ_YOLO_ = 1.0 are hyper-params set to balance number loss and YOLO loss.

### Model training

We use Python as the programming language, Pytorch [31] as the deep learning framework, and the AdamW optimizer. Because the YOLO X model is large, the original YOLO X trained on NVIDIA GeForce RTX 3090, the improved YOLO X models with Mixed Precision are trained on 2080Ti.

The models used pre-trained YOLOX-L. Because the YOLO head was modified heavily, the training was divided into two stages. In the first stage, the YOLO head was trained, and in the second stage, the whole model was trained. The specific train parameters are shown in Table 2.

**Table 2:** Training parameters of YOLO POD

Stage	Epochs	Learning rate	Gamma	Batch size
1	5	0.001	0.92	8
2	55	0.0001	0.90	2

In Stage 1, training YOLO POD head. In Stage 2, training the entire YOLO POD

## Results and discussion

### Comparison with other object detection models

We compared YOLO POD with some mainstream and classic models, including Mask R-CNN [32], Swin Transformer [33], YOLO V4 [23], and YOLO V5 [29]. The result is shown in Table 3. Compared to YOLO X, the R^2^ of YOLO POD improved by 0.049, reaching 0.967, while MAE, MAPE and RMSE all decreased significantly. For soybean counting, YOLO POD completely outperforms existing models, achieving high accuracy and low error.

**Table 3:** Comparison of the accuracy of different object detection networks

Models	R^2^	MAE	MAPE	RMSE
Mask R-CNN	0.824	16.0	29.6%	25.0
Swin-S Mask R-CNN	0.894	10.1	18.3%	18.0
YOLO V4	0.914	11.5	23.1%	17.5
YOLOV5-L	0.921	11.1	17.8%	14.0
YOLOX-L	0.918	8.72	17.5%	14.4
YOLO POD	**0.967**	**4.18**	**10.0%**	**6.48**

All the methods are tested at 1024 × 1024 resolution. Bold text indicates the best results.

In addition, compared with the original Mask RCNN, using Swin Transformer as the backbone, the results were significantly improved. On many datasets, the best results are achieved by using Swin Transformer as the backbone [33], Combining YOLO POD with Swin Transformer might achieve a better result. However, in order to achieve the best results, the transformer structure requires more data than CNN [34], while the labeling of dense objects like pods is expensive and difficult. Moreover, Swin Transformer is computationally expensive, which limits its deployment and application.

The results of the YOLO series models are generally better compared to Mask RCNN. YOLO series models are designed for the object detection task, while Mask RCNN is designed for the instance segmentation task. This suggests that for pod counting, segmentation is unnecessary and may affect the performance of the model.

**Fig. 6:**
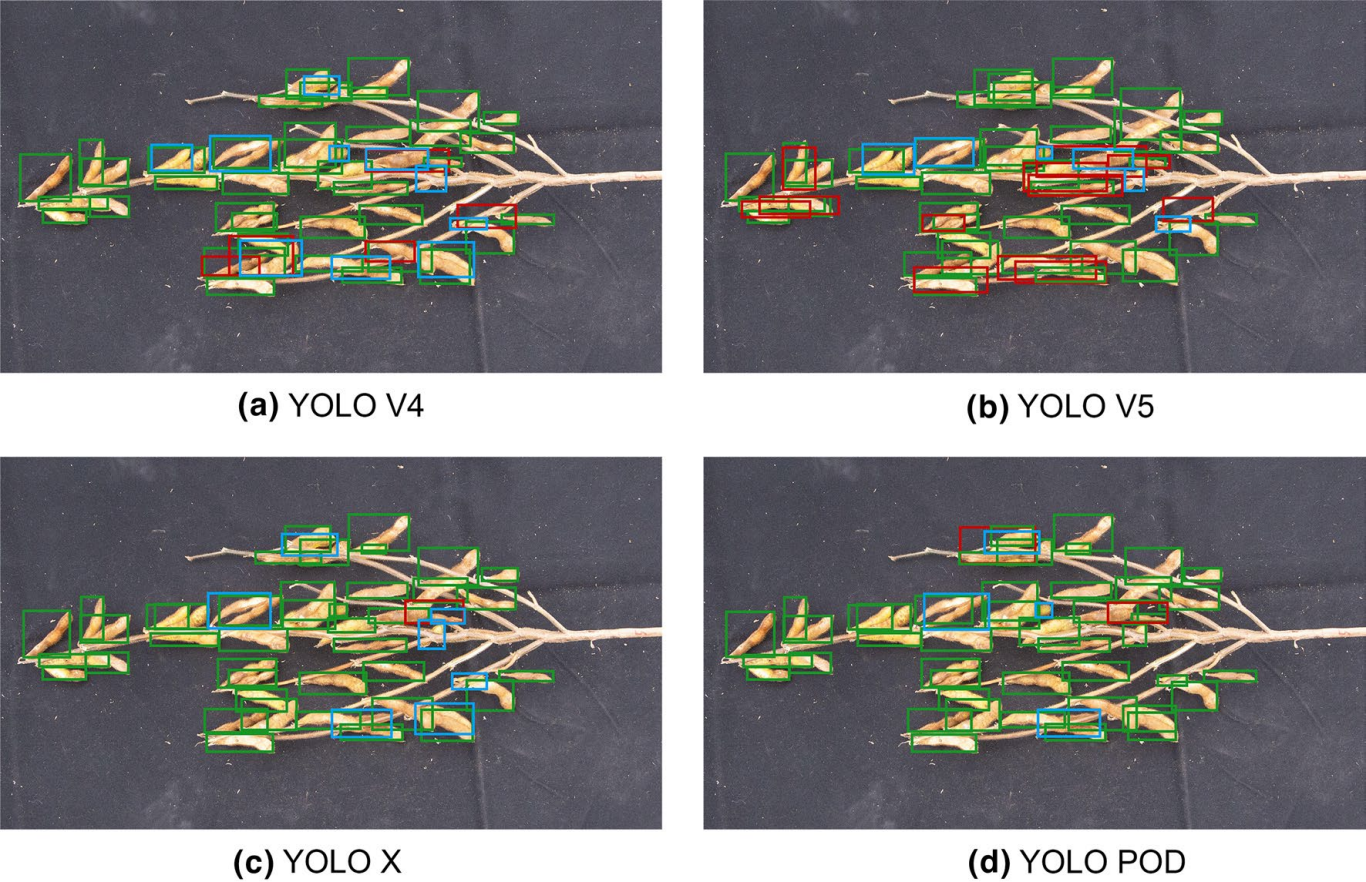
Example of the detection results of the YOLO series on the Renshou 2021 dataset. The green boxes represent the pods that were correctly detected, the red boxes represent the incorrect results, and the blue box represents the missing pods

Some of the detection results of the YOLO series are shown in Fig. 6. In Fig. 6, the green boxes represent the pods that were correctly detected, the red boxes represent the incorrect results, and the blue boxes represent the pods that were missed. As it can be seen from the graph, YOLO V5 has more misidentified results, and YOLO X has more unidentified pods. The YOLO POD has the least missed detection and false detection, and its effect is the best.

### Comparison of model detection speed

In order to compare the detection speed of YOLO POD and other models, we tested different models on the Renshou 2021 and Renshou 2022 dataset, and the specific results are shown in Table 4. For YOLO POD, when training, the parameters is 78.6 M and the FLOPs (floating point operations, used to measure the computational complexity of the model, the smaller the better), is 445.8G, but in inference, the Number Prediction Module is not used, the Parameters is 54.2 M and FLOPs is 394.9G.

**Table 4:** Comparison of detection speed of different models

Models	Inference speed	Parameters	FLOPs
Mask R-CNN	15.0 s	43.8 M	499.8G
Swin-S Mask R-CNN	9.81 s	69.1 M	723.5G
YOLO V4	0.419 s	63.9 M	362.1G
YOLOV5-L	0.413 s	46.5 M	292.5G
YOLOX-L	0.454 s	54.2 M	397.8G
YOLO POD	0.462 s	54.2 M	394.9G

All the models are tested at 1024 × 1024 resolution, and batch = 1 on 2080Ti

Mask R-CNN and Swin Transformer are mainly for instance segmentation tasks, so the model is large and the inference speed is slow. Among them, although the Swin Transformer has a good detection effect, the model speed is slow and the GPU requirements are high.

In the YOLO series, YOLO X is slower than YOLO V4 and YOLO V5, mainly because of the design of the model. Compared to YOLO X, the parameters of YOLO POD do not change significantly, while FLOPs have decreased, the decrease in FLOPs is mainly due to the introduction of SPPF, which reduces the calculation. In terms of inference speed, YOLO POD is slower than YOLO X. The increase time is mainly from the CBAM. But YOLO POD is only 0.08 s slower than YOLO X, YOLO POD greatly improves the accuracy of detection with a slight increase in inference time.

### Comparison with previous works

Table 5 contains our and previous methods for counting soybean pods. The AP_50_ is calculated with reference to the COCO [15]. Riera et al. [12] input three images into RetinaNet [35] for detection, thus estimating the number of pods. Yang et al. synthesized a pod dataset for training Swin Transformer, and use 200 real soybean plant images to evaluate the detection effect [14].

**Table 5:** Comparison with other methods in pod detection and counting

Methods	R	AP_50_	References
RetinaNet + Multiview Image	0.711	-	[12]
Swin transformer + Synthetic Dataset	-	0.800	[14]
Ours	0.983	0.839	–

The correlation between the predicted and actual values of our method is much higher than that of Riera et al., already available for practical application in production. In terms of detection effect, the AP_50_ of our method is slightly better than that of Yang et al. But considering the large size and slow speed of the Swin Transformer, our method is more valuable in practical application.

### Validation of improvement measures

**Fig. 7:**
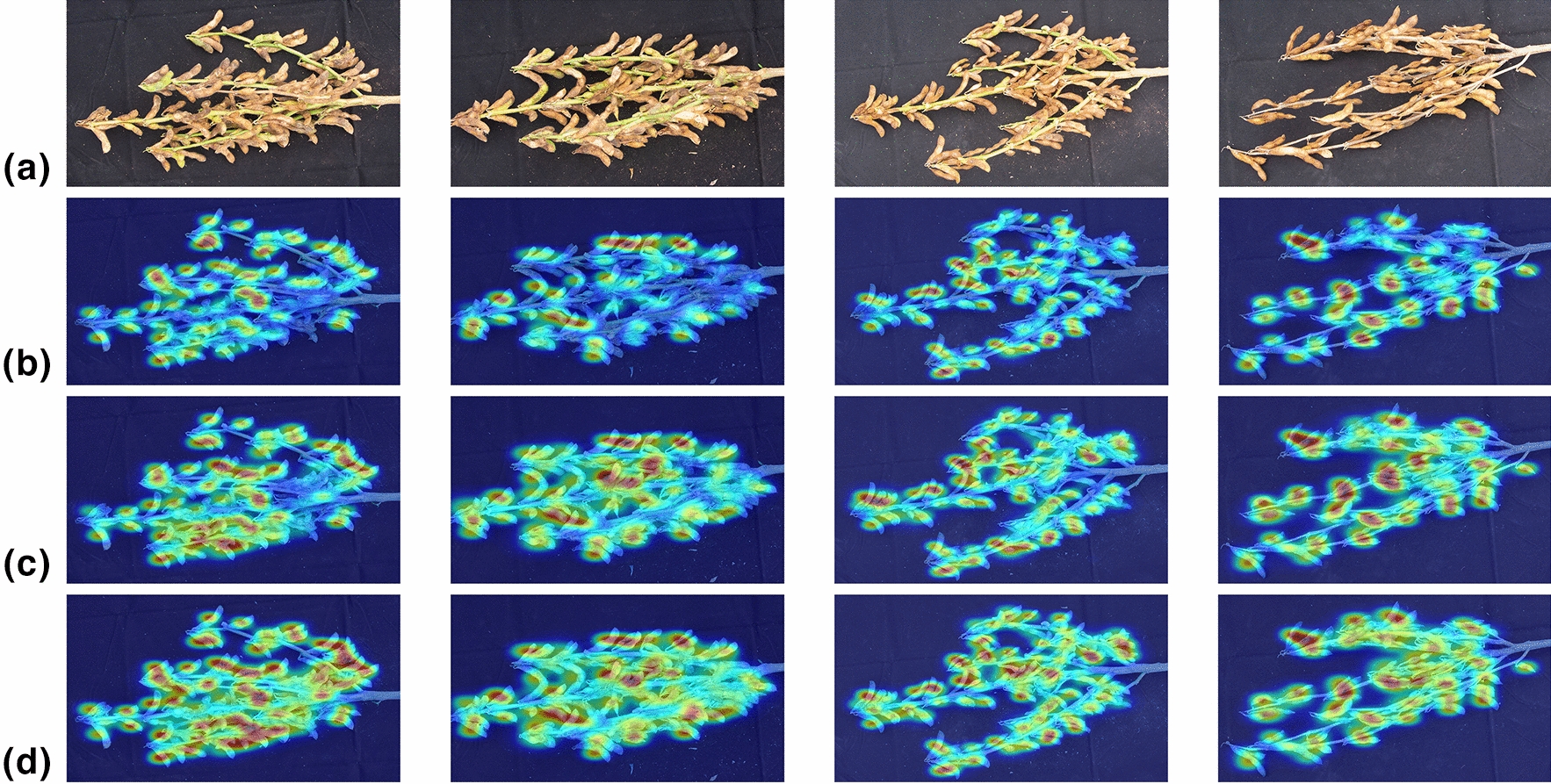
Examples of heat maps of different models: **a** input images; **b** YOLO X; **c** YOLO POD without Number Prediction Module; **d** YOLO POD

The heat map shows which areas of the image are mainly used by the model when recognizing, the more the model focuses on a region, the higher temperature. Figure 7 shows examples of heat maps for different models.

As can be seen in Fig. 7b, YOLO X focuses on fewer areas, mainly on the intact, unobstructed pods. After improvements, the model identified more pods, this is mainly attributed to the appropriate data augmentation strategy and CBAM. The data augmentation provides more samples for the model, and the CBAM can emphasize important features and suppress unnecessary ones [30], it makes the model pay more attention to the area with pods.

With the introduction of Number Prediction Module, the region of interest of the model is further expanded. The information of the heavily overlapping regions is also noticed and used by the model. In areas where the pods overlap heavily, the bounding boxes are close together and overlap each other. In the model, images are down-sampled by 8×, 16 × and 32× some bounding boxes might be ignored when calculating losses. The Number Prediction Module predicts the number of pods in the whole image based on the extracted features, without relying on the labeled bounding boxes, this makes the model more attentive to overlapping regions. Additionally, due to the change of the loss function, the weight of YOLO loss is reduced, preventing the model from overfitting.

To further verify the effectiveness of the Number Prediction Module, we designed a series of experiments. We additionally calculated Precision and Recall. Precision is the percentage of correctly identified pods among the prediction result, the higher the Precision means the higher the accuracy of the model. Recall is the percentage of correctly identified pods among all labeled pods, the larger Recall indicates the higher integrity of the segmented pods. And here we calculate the AP_50_ according to the VOC dataset [36], which is different from that in 3.3. The results are shown in Table 6.

**Table 6:** Comparison of different ways of using the Num Prediction Module

Num Prediction Module	target	AP_50_	Recall	Precision	R^2^	MAE	MAPE	RMSE
×	-	85.98	84.44	**82.34**	0.9533	6.170	13.72%	9.945
√	Random	86.32	84.97	82.06	0.9594	5.271	11.12%	8.885
√	Number of pods	**87.41**	**86.57**	80.38	**0.9666**	**4.184**	**10.04%**	**6.477**

Bold text indicates the best results.

When the optimization objective of the Number Prediction Module is a random number between 0 and 299, the model cannot learn any meaningful information through the Number Prediction Module. But the AP and Recall are slightly improved, the loss term brought by Number Prediction Module reduces the overfitting of the model.

When the optimization objective of the Number Prediction Module is the number of pods in an image, AP improves by 1.43 and Recall improves by 2.13, which indicates that the Number Prediction Module helped the model to identify more pods. Furthermore, the R^2^ between the predicted number and the manually counted number increased by 0.0133, and the MAE, MAPE, and RMSE decreased by 32.2%, 27.5%, and 34.8%, respectively. The information learned through the Number Prediction Module effectively helps the model to improve the accuracy of the pod counts.

## Conclusion and future work

We propose a soybean pod counting model based on the YOLO framework. Experimental results show that a suitable auxiliary task can help improve the main task. By improving the model structure and multi-tasking the design, fast and accurate counting of soybean pods were achieved, and the proposed model completely outperforms existing object detection networks. After harvest, simply take a picture of soybean plants with black background, and the YOLO POD can quickly and accurately estimate the number of pods. YOLO POD can replace manual labor, and greatly improve the efficiency of breeding. Additionally, we believe that our state-of-the-art results can inspire other dense object counting tasks.

It is very convenient and efficient to use the unmanned vehicles to take images and then estimate the yield from the images. But unlike rice and wheat ears, soybean pods are not located at the top of the plant, so the number of pods cannot be estimated from the field images taken by the unmanned vehicles. Our practice is to take images indoors after harvesting soybeans, which limits the application of YOLO POD. To enable YOLO POD to be used in the field, mobile automatic imaging devices need to be developed in the future.

Another limitation of this study is the dataset. Although this paper collected thousands of soybean images from two regions, this dataset is not large and rich enough compared to the Global Wheat Head Dataset [37]. In the future, more images of soybeans from different regions and countries need to be collected to build a larger dataset and enhance the generalization ability and generalizability of the model. In addition, knowledge distillation of the model needs to be attempted to further compress the model and improve the model inference speed, so that the model can be deployed on more devices. This would be a fruitful area for further work.

## Supplementary Information

**Additional file 1.** Soybean variety names and sources

## Data Availability

The datasets generated and analysed during the current study are available in the Google Drive repository, https://drive.google.com/drive/folders/1-Ouj8fFG_owOnJtDDGBQ29_gDyCUdu93?usp=sharing.
