# Correction: YOLO POD: a fast and accurate multi-task model for dense Soybean Pod counting

**DOI:** 10.1186/s13007-023-01013-1

**Published:** 2023-05-12

**Authors:** Shuai Xiang, Siyu Wang, Mei Xu, Wenyan Wang, Weiguo Liu

**Affiliations:** 1 College of Agronomy, Sichuan Agricultural University, 211-Huimin Road, Wenjiang District, Chengdu, 611130 People’s Republic of China; 2 Key Laboratory of Crop Ecophysiology and Farming System in Southwest China (Ministry of Agriculture), Sichuan Engineering Research Center for Crop Strip Intercropping System, Sichuan Agricultural University, Chengdu, 611130 People’s Republic of China

**Correction: Plant Methods (2023) 19:8** **https://doi.org/10.1186/s13007-023-00985-4**

In the original publication of the article [1] Figures 2, 3 and 4 were wrongly numbered and the citations were incorrect. The Fig. 4 should have been Fig. 2; Fig. 2 should have been Fig. 3; Fig. 3 should have been Fig. 4 as shown below. The placement of figures and their citations were corrected. The original article has been corrected.

**Fig. 2:**
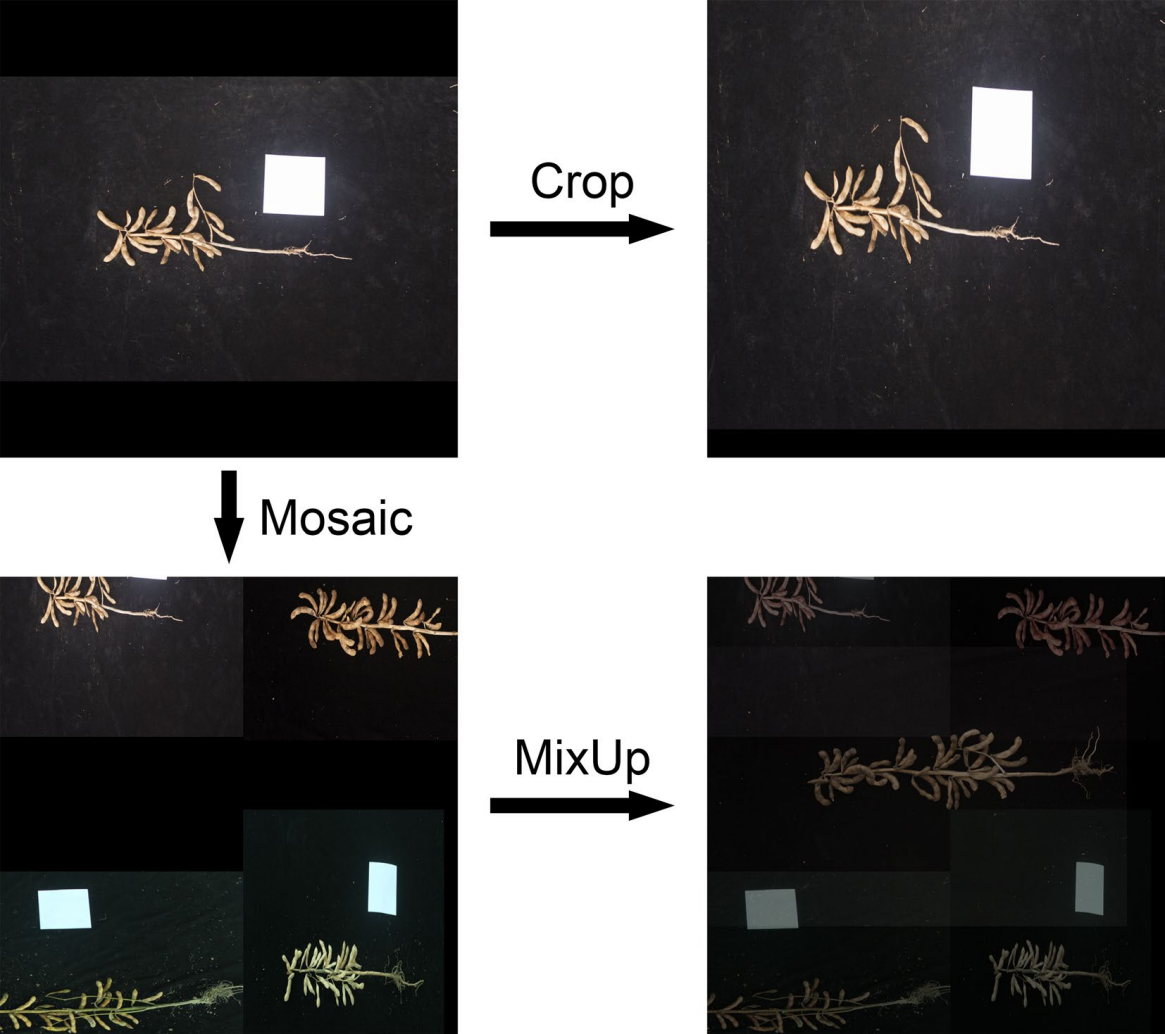
Illustration of YOLO POD’s image augmentation pipeline

**Fig. 3:**
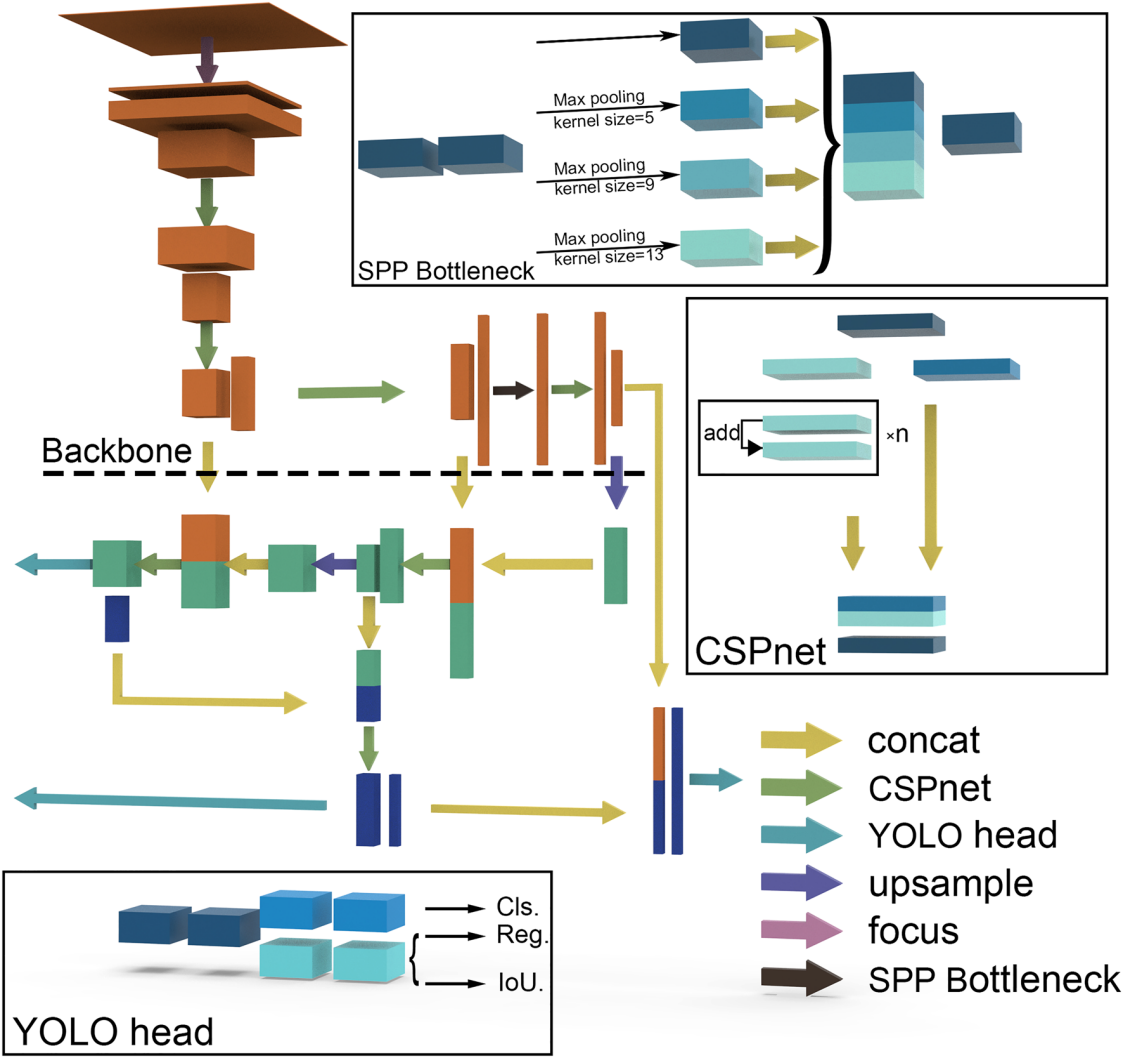
Illustration of the overall structure and sub-modules of YOLO X

**Fig. 4:**
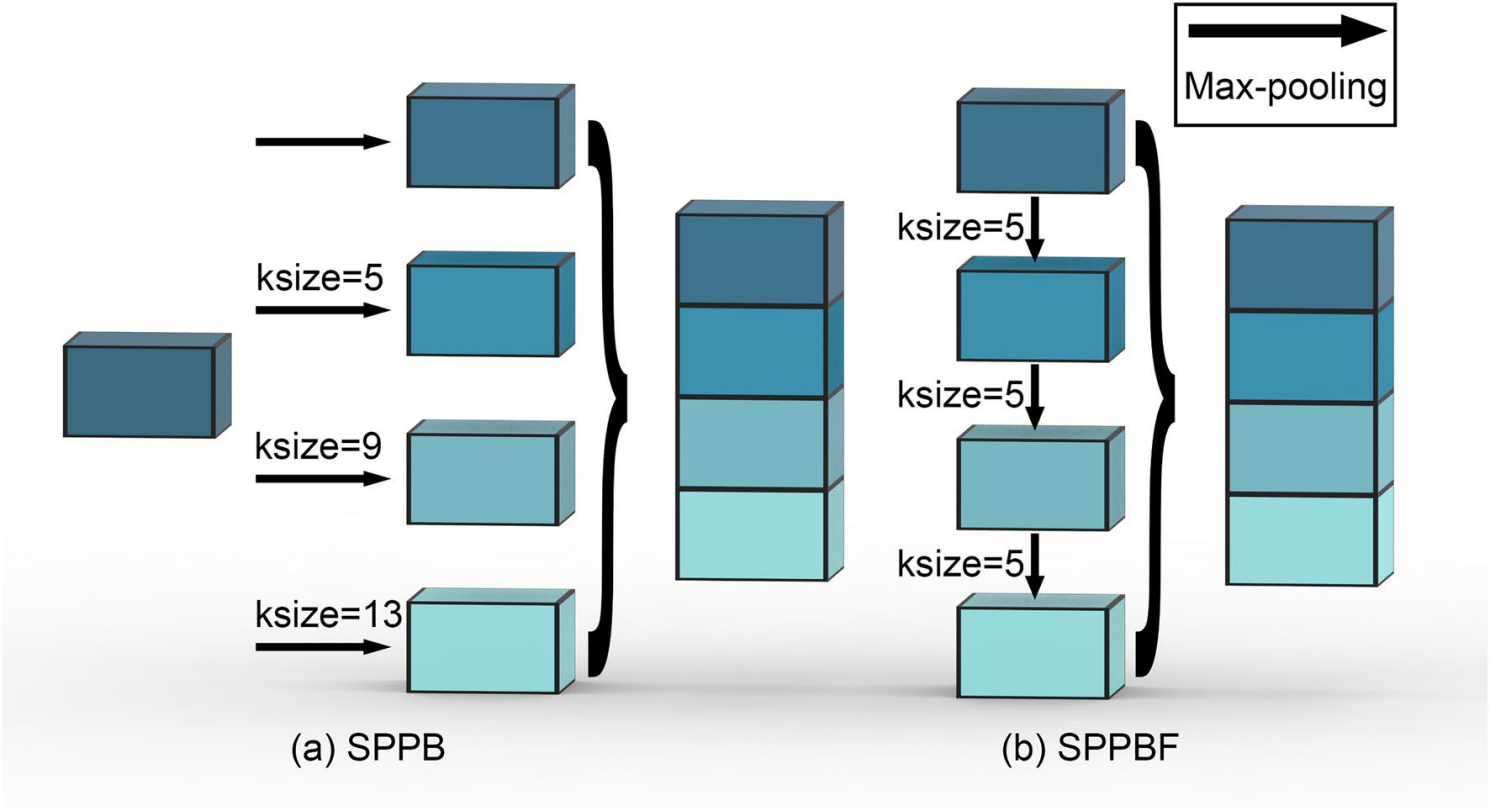
Illustration of the difference between Spatial Pyramid Pooling and the Spatial Pyramid Pooling-Fast. SPPB uses 3 pooling layers with different kernel-size, while SPPBF uses 3 consecutive pooling with kernel-size=5
